# Keeping Hope Possible Toolkit: The Development and Evaluation of a Psychosocial Intervention for Parents of Infants, Children and Adolescents with Life Limiting and Life Threatening Illnesses

**DOI:** 10.3390/children8030218

**Published:** 2021-03-12

**Authors:** Jill M. G. Bally, Meridith Burles, Shelley Spurr, Lorraine Holtslander, Heather Hodgson-Viden, Roona Sinha, Marcelline Zimmer

**Affiliations:** 1 College of Nursing, University of Saskatchewan, Saskatoon, SK S7N 5E5, Canada; meridith.burles@usask.ca (M.B.); shelley.spurr@usask.ca (S.S.); lorraine.holtslander@usask.ca (L.H.); 2 Faculty of Health Sciences, University of the Witwatersrand, Johannesburg 2193, South Africa; 3 College of Medicine, University of Saskatchewan, Saskatoon, SK S7N 5E5, Canada; heatherhodgson.viden@usask.ca (H.H.-V.); roona.sinha@usask.ca (R.S.); 4 Ronald McDonald House Saskatchewan, Saskatoon, SK S7N 0K4, Canada; mzimmer@rmh.sk.ca

**Keywords:** intervention, psychosocial, hope, parents, life limiting illness, life threatening illness, children, complex healthcare needs

## Abstract

Background: Globally, many infants and children are diagnosed with illnesses that impose limitations on their well-being and life course trajectory. Children’s care becomes the central focus of family life. Inadequate support for parents is detrimental to their well-being and management of their child’s care and support needs. Methods: The second phase of this evaluation study followed a quasi-experimental crossover design to test a theory-based psychosocial intervention, the Keeping Hope Possible Toolkit. Fifty-nine participants were randomly assigned to one of two sequence groups, with measures of hope, feelings of control, distress, and uncertainty completed pre- and post-intervention, and at a three-month follow-up. Qualitative interviews sought to assess participant experiences with the intervention, along with acceptability and feasibility. Results: Significant influence on parental distress was found, and the qualitative findings reveal benefits of the intervention for parental wellbeing. The intervention effectively offered practical and emotional support to diverse family caregivers. Conclusions: The evidence-informed KHP intervention can be used by healthcare providers to intervene with family caregivers to support their dynamic emotions including hope, need to live in the moment and remember self, and social preferences. In doing so, parents’ critical caregiving activities can be sustained and their child’s health and wellbeing optimized.

## 1. Introduction

Globally, many infants, children, and adolescents are diagnosed with illnesses that impose limitations on their well-being and life course trajectories. The exact number of diagnoses is difficult to determine because of the complex array of such conditions; however, there are approximately 2400 deaths annually in Canada [1], many of which relate to illness and medical complications. More specifically, in Saskatchewan, Canada where the research was conducted, over 200 children with complex treatment needs receive diagnoses of life limiting and life threatening illnesses (LLIs, LTIs), and specialized pediatric palliative care each year (H. Hodgson-Viden, personal communication, June 2020). As medical research advances treatment and health care, many young people live with life-limiting (LLI) and life-threatening illness (LTI) [2].

Often, management of such illnesses requires complex medical care and a multitude of therapies [2]. Consequently, children’s care and support can become the central focus of the family’s life, with daily activities revolving around management of symptoms and/or illness-imposed limitations along with basic physical, emotional, and social needs [2,3]. Parents especially might find their daily lives centered on supporting and caring for their child through navigation of medical appointments and care activities along with advocacy, transportation, education, and relationship management [2]. Consequently, many parents experience significant disruption to prior roles and activities, including those related to work and social life [3,4,5,6]. Challenging emotions are also common, with parents expressing fear for their child’s well-being and life course, shock, sadness, and guilt [2,7,8]. Persistent worry and the uncertainty of the future can leave parents feeling helpless and as if they have lost control, which can be associated with anxiety and depression [2,9,10].

While navigating the complex circumstances related to a child’s illness, parents can be in grave need of support. Specifically, many parents sacrifice their own well-being and prior roles to focus on their child [11]. Furthermore, some parents struggle to obtain assistance, either because they are unsure where to find support or do not want to be perceived as an inadequate parent or burden to others [10]. Social interactions can also be difficult and exhausting to negotiate [12], and parents may not have time to socialize with extended family or engage in leisure activities [13], thus limiting their support networks. The need to transport medical equipment and a child’s medical fragility also hinders activities outside of the home [3]. Therefore, some parents might opt to minimize social contact, whereas others find themselves socially isolated due to the circumstances of their child’s condition, which can contribute to unmet support needs. 

Given the significant disruption to family life brought about by LLI and LTI in a child, it is crucial to support the whole family unit, especially parents, as they navigate their child’s care and establish new routines [3,5,11]. Inadequate support for parents is detrimental to their well-being and management of their child’s care and support needs [3,11]. Parents who achieve a sense of normality and control in their daily lives and develop hopefulness will be better equipped to endure the challenging circumstances associated with their child’s condition [11,12]. In doing so, they can provide a steady presence to their child and support development of effective coping strategies to quell fears and anxieties related to medical treatment. However, achieving a sense of normality and hopefulness while navigating complicated emotions and uncertainty of the future, along with instrumental tasks, can be difficult [11,12]. Thus, it is imperative to develop and make available effective supports for such parents that attend to their psychosocial well-being as a component of family centered care of children with LLI and LTI. 

Despite that existing literature highlights the substantial support needs of parents of children with LLI and LTI, only a few psychosocial interventions have been developed for use with this population. Namely, five psychosocial interventions aimed at supporting parents or family caregivers of children with chronic and terminal illnesses were identified in a review of recent research [14,15,16,17,18]. These interventions varied in terms of format and target population to some extent, but all focused on supporting psychosocial well-being in parental caregivers of children with some form of LLI and LTI. Three interventions involved in-person sessions, with variation in the number of sessions and activities involved. For example, one intervention engaged parents of children with cerebral palsy in a strength-based focus group aimed at supporting quality of life [14]. Participation was shown to benefit parental coping skills immediately after, but long-term impacts were not assessed. Another intervention involved music therapy sessions with parents of children whose LTI was in the terminal stage. These sessions were found to support positivity and hope, along with improved quality of life and communication within families [15]. Finally, a third intervention adopted a high-intensity approach, with daily sessions over two weeks or a month implemented with parents of children with cancer to promote positive emotional responses and coping [16]. Overall, participation in the intervention had positive outcomes for parent-child units in comparison to those receiving standard care. 

Meanwhile, two other interventions described in the existing literature were offered on a more flexible, individual basis. Specifically, one intervention adopted an innovative Narrative e-Writing approach aimed at supporting parents’ psychosocial well-being through web-based sessions facilitated by a therapist [17]. This four-week writing intervention is currently being evaluated, but shows promise in supporting parental quality of life and hope among other outcomes. The format makes this intervention accessible and cost effective. Another study evaluated use of a resource booklet as a psychosocial intervention for parents caring for children with LLI and LTI, with the aim of determining its value in planning future care [18]. While the resource booklet was deemed useful by many participants, barriers within the health care system were identified, which were thought to hinder access to appropriate care, communication, and effective use of the booklet. As such, evaluation of the resource booklet revealed that it would need to be accompanied by improvements in support and resources to have significant benefits for parents. While these few interventions exist in various stages of development, few are geared towards a variety of childhood illnesses, and many are tethered to institutional programs and may not be used outside of place in which the intervention is administered. Accordingly, there is a need for development of psychosocial interventions that promote the holistic well-being of parents and other family caregivers involved in care and support of children with LLI and LTI in ways that have broad appeal and do not add additional burden to already complex schedules. 

Given the extensive impacts that LLI and LTI in children can have on parents and families, it is essential to ensure availability of effective support to sustain family caregivers’ important role in their children’s care. Psychosocial support that promotes holistic well-being of family caregivers can facilitate navigation of complicated emotions along with complex care and therapy activities. Processes that support hope as a psychosocial resource have been found to be particularly valuable to family caregivers [11,19,20]. Accordingly, to meet the existing need for enhanced support of family caregivers, our research team developed a psychosocial intervention for parents of infants, children, and adolescents called the Keeping Hope Possible (KHP) Toolkit. The initial iteration of the intervention was based on two prior phases of research including a qualitative study of parents’ experiences when a child has cancer [19] and a Delphi study that collected data from parents, pediatric health care providers, and support providers [20]. 

The KHP Toolkit was developed as a self-administered intervention with emphasis placed on convenience, flexibility, and adaptability to diverse experiences. The KHP Toolkit includes four main sections with two sub-sections with practical information and planning tools that are interspersed with reflective activities, with the aim to foster development of instrumental and psychosocial coping strategies. For example, some activities include documenting sources of support and meaningful information, while others focused on mindfulness, living in the moment, and celebrating every milestone. After pilot-testing the initial version [21], the KHP Toolkit was refined for evaluation with a larger sample of family caregivers of children who had been diagnosed with an LLI or LTI within the preceding year. The purpose of this research, as described here, was to assess whether the KHP Toolkit was feasible, appropriate, and effective in fostering hope and self-efficacy while reducing uncertainty and distress. The research was guided by the research questions: (a) what are the levels of hope, feelings of control, distress, and uncertainty in parents of children who have been diagnosed with LLIs or LTIs?; (b) does the theory-based psychosocial hope intervention influence parents’ hope, feelings of control, distress, and uncertainty?; and, (c) is the existing intervention and the research process acceptable to, and feasible for, parents of children with LLIs and LTIs?

In this article, the KHP Toolkit development process is described and the findings of the qualitative and quantitative components of the evaluation study are reported. The value of psychosocial interventions that support family caregivers as they care for children with LLI and LTI is described. Additionally, recommendations for development and refinement of such interventions, as well as for future research.

## 2. Materials and Methods

Following the pilot-test, the KHP Toolkit was deemed to be appropriate with minor revisions and inclusive of valuable information and activities that promote coping and keeping hope possible. Accordingly, the intervention showed potential to enhance family centered care in pediatrics by offering a psychosocial support intervention that is convenient and adaptable to family caregivers’ individual circumstances and preferences. The research described herein is the second part of a two-phase, quasi-experimental, intervention study.

**Figure 1:**
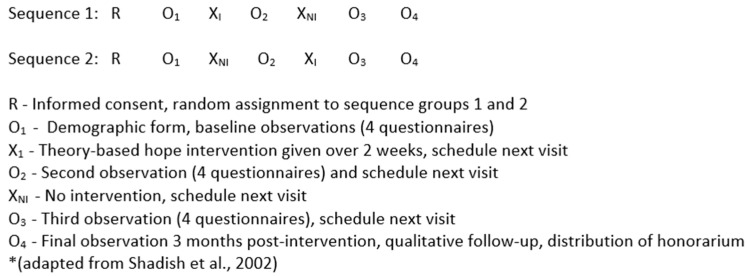
Crossover Design and Research Process.

The second phase of the research sought to evaluate the KHP intervention with 55 to 60 parents of children with LLIs and LTIs. Specifically, the refined KHP intervention was tested using a quasi-experimental crossover design [22] as outlined in Figure 1.

Participants were randomly assigned to one of two sequence groups, with measures of hope (HHI) [23], feelings of control (GSES) [24], distress (K6) [25], and uncertainty (PPUS) [26] collected pre- and post-intervention, and at a three-month follow-up to evaluate the effectiveness of the intervention. Additionally, analyses comparing pre-intervention, post-intervention, and follow-up responses were conducted. Qualitative interviews were conducted to assess participant experiences with, and the acceptability and feasibility of the intervention, as well as to close the research process with each participant. 

### 2.1. Measures

The results reported here are derived from a demographic questionnaire along with four scales: The Herth Hope Index (HHI) [23]; General Self Efficacy Scale (GSES) [24]; Kessler 6 Psychological Scale (K6) [25]; and the Parental Perception of Uncertainty Scale (PPUS) [26]. Internal reliability was examined for the four scales using Cronbach’s alpha coefficient. A detailed description of the study measures is published elsewhere [21]. The coefficients for each of the four scales were reliable measures of the variables of interest and are outlined in Table 1.

### 2.2. Interviews

In addition, one face-to-face, open-ended, audio-taped interview was completed with those participants who had completed the quantitative measures at T1, 2, 3, and 4, and agreed to participate. A flexible interview guide provided a framework for each interview. The questions provided a guide for the interviews but were not always posed in order, or at all, depending on the participant’s responses. Questions sought to gain an understanding of the participants’ experiences with, and opinions of the KHP Toolkit such as: How did you use the KHP Toolkit?; what did you like best about the KHP Toolkit?; what did you like least about the KHP Toolkit?; what did you find most helpful about the intervention?; and, if you could improve this intervention, how would you do so?

### 2.3. Sample

Participants from Phase 1 were excluded to avoid any potential bias or contamination in Phase 2. To establish sample size, it was determined that by assigning 25–30 participants to each group, the minimum sample size requirements would be met for repeated measures ANOVA, as the sample size in each cell will exceed the number of dependent variables. Therefore, a sample of 55–60 parents of children with a LLI or LTI was sought and included: Parents or other caregiver (e.g., grandparent) of any age or gender who were the primary care provider for a child between three months and 14 years of age. To be included, the child needed to be within 12 months of diagnosis with any LLI or LTI, and under the care of pediatric oncology services or pediatric palliative care in one provincial healthcare system. As well, participants needed to be English speaking and freely able to provide informed consent. Parents were recruited with the assistance of recruitment supporters (nurses, social workers, and physicians) using purposive sampling, aiming for a diverse sample to capture a variety of experiences (e.g., different diagnoses, times since diagnosis, genders) and comprehensive perspectives. Consecutive sampling was also used such that participation was offered to all eligible and interested caregivers and none were turned away. Additionally, both parents from the same family were invited to participate, and when this was the case, they were placed in the same random group as one another to avoid cross-contamination.

### 2.4. Data Collection Procedures

Arrangements were made to meet potential participants in the hospital setting, with written informed consent being obtained prior to data collection. Participants were randomly assigned to one of two intervention sequence groups. Randomization was achieved by placing the data collection tools and a KHP Toolkit in numbered envelopes (one = immediate intervention group; two = intervention after two weeks), and shuffling the envelopes. When a participant agreed to participate in the study, the envelope at the top of the pile was selected for that participant. Both groups of participants completed a demographic questionnaire and four measures including the HHI [23], GSES [24], K6 [25], and PPUS [26] at Time 1 during the first meeting. Sequence group 1 received the intervention immediately after completing the measures, whereas sequence group 2 initially had no intervention. At Time 2 (approximately 2 weeks after Time 1), sequence group 1 had used the intervention for two weeks, whereas sequence group 2 began to use the intervention. Both groups completed all four measures again at Time 2, with group 2 completing measures before beginning the intervention. At Time 3 (approximately 2 weeks after Time 2), both groups had completed the intervention sequence and completed all four measures again. Approximately three months after Time 1, follow-ups were completed with all participants (Time 4) by administering the same measures along with a brief qualitative interview that sought to capture participants’ subjective experiences of using the intervention. Some participants completed questionnaires on paper, whereas some completed them electronically using REDCap^®^ (Research Electronic Data Capture), a secure, web-based software platform designed to support data capture for research studies. In most cases, Times 2, 3, and 4 were done electronically. Interviews took place in a location of each participant’s preference, and most were audio-recorded for accuracy. Detailed notes were taken in two cases, and three other participants responded to the interview questions by email. The interviews also allowed for debriefing and closure of the research process. 

### 2.5. Data Analysis and Interpretation

All quantitative analyses were conducted using IBM^®^ SPSS^®^ (Version 26) predictive analytics software. Analyses comparing pre-intervention, post-intervention, and follow-up responses were conducted using a 2 (intervention sequence, between subjects) × 4 (time, repeated measures) repeated measures ANOVA. 

The qualitative interviews were transcribed verbatim and analyzed in alignment with interpretive description methodology [27]. NVivo11™ qualitative data analysis software was used to organize and store the data during data analysis. Analysis began with consideration of what the interview transcripts meant individually and how they related to one another, followed by identification of prominent experiences and processes, and prioritizing or sequencing the key conclusions [27]. Subsequently, linkages and patterns were identified and synthesized allowing for the re-contextualizing of data so that findings could be applied to other contexts [27]. Coding identified categories, patterns, and relationships amongst the data. Furthermore, constant comparative analysis was applied to examine and compare data with all other pieces of data to continuously consider relationships, similarities, and differences throughout the analytical process [27,28]. As data analysis progressed, two members of the research team met with two research assistants regularly to discuss and to reflect on emerging codes and themes. Any discrepancies were discussed and applicable data re-analyzed such that there was a true representation of participant experiences based on our interpretation of the data. Once all codes were represented by each category, the data was presumed to have captured the perspectives of all participants. 

According to Thorne [27], evaluation of credibility and rigor in Interpretive Description research consists of epistemological integrity, representative credibility, analytic logic, and interpretive authority. Epistemological integrity of the entire study was based on a pragmatic approach that incorporated qualitative and quantitative data. Representative credibility emerges from the detailed description of the sample characteristics and data collection and analysis, along with triangulation of the data sources. Analytic logic was ensured by transparent decision-making, employing a carefully selected team of researchers who were experienced in pediatric/family care, research, and education, keeping an audit trail, and representing the data using direct quotes. Immersion in the data, staying open, and forming our interpretations based on the participants’ experiences ensured interpretive authority. Together, the quantitative and qualitative findings enabled evaluation of the effectiveness, feasibility, and acceptability of the KHP Toolkit.

### 2.6. Ethical Considerations

The research began following ethical approval from the University of Saskatchewan Behavioural Research Ethics Board, and operational approval from the related health authority. For every participant, informed consent was obtained by a research assistant or member of the research team once they received details of the study and agreed to participate. Specifically, potential participants were informed that participation was voluntary and there were no guaranteed benefits from enrollment. Given the crossover design [22], all participants received the intervention, ensuring ethical conduct of interventional research. Participants were informed that answering survey and interviews questions could elicit an emotional response and they could omit answering any questions they chose. Although it was not necessary, research team members were prepared to contact support services for participants who became upset, with their permission. Consent forms with the participant names and demographic forms were stored separately from participants’ data. All findings from this study are reported in an aggregate format or anonymous form so that no individual participant can be identified.

## 3. Research Findings

### 3.1. Quantitative Sample and Findings

A total of 58 participants completed the measures of their HHI [23], GSES [24], K6 [25], PPUS [26]. Data were collected pre- and post-intervention for both groups (T1 *n* = 58, T2 *n* = 50, T3 *n* = 45, respectively) and at a three-month follow-up (*n* = 26) to evaluate the effectiveness of the intervention. Due to the complex circumstances of families including the death of a child, travel for treatment, and a busy schedule, some participants were lost to attrition. The sample had a representation of male (*n* = 12) and female (*n* = 46) participants ranging in age from 20 to 53 years (M = 34.63) and included mothers (*n* = 44), fathers (*n* = 12), grandparents (*n* = 1), and foster parent (*n* = 1). Demographic data are reported in Table 2. 

The children in this study represented a variety of ages (under 3 years, *n* = 27; 3–6 years, *n* = 7; 7–10 years, *n* = 7; and 11–14 years, *n* = 5) and a variety of LLIs and LTIs such as aplastic anemia, genetic/chromosomal anomalies, cerebral palsy, leukemia/lymphomas and a variety of other cancers, leukodystrophy, spinal muscular atrophy, and severe stroke.

Data analysis showed that the participants had increased hope and self-efficacy and decreased uncertainty and psychological distress after the intervention. Specifically, the scores improved beyond the baseline level for every measure including the HHI [23] (37.31–37.96), GSES [24] (32.29–32.58), K6 [25] (8.54–5.79), and PPUS [26] (89.67–87.21). In the overall comparison of the four times (T1, T2, T3 and T4), the repeated measures ANOVA indicated that T1 was statistically significantly different from the other three times points (T2, T3, T4) in terms of the psychological distress (K6) [25] (F = 6.407, *p* = 0.001). However, the survey questionnaire did not show significant changes across T1, T2, T3, and T4 for the other three measures (HHI [23], GSES [24], PPUS [26]), despite that some differences are evident. Additional analyses indicated no statistically significant differences between the two groups in terms of demographics. The means for each measurement time are reported in Table 3. 

### 3.2. Qualitative Sample and Findings

Of those included in the quantitative study, 29 family caregivers participated in the qualitative face-to-face, audio-taped, open-ended evaluation interviews and three participants responded in email format, for a total of 32 respondents (*n* = 32). Each interview lasted between eight and 50 minutes. Analysis of the transcribed interviews permitted enhanced understanding of the participants’ experiences during their participation in this research which were related to, and integrated with what participants saw as the benefits of using the KHP Toolkit. In addition, participants discussed potential improvements that could be made to the KHP Toolkit. 

During participation, parents were caring for children with various illnesses and complex medical needs but shared similar experiences. Generally, parents described their experiences as chaotic, overwhelming, and one in which there was information overload, particularly at the time of diagnosis. For example, one participant stated ‘’when you’re in the thick of it, like in the early dark days of the journey, you’re in a bit of a survival mode.” Another participant concisely described the experience of caring for a child with serious illness when she stated,

“I’ve always understood that you can’t define normal. I’ve always been respectful of that but adjusting to our *new normal* has been hard because every sniffle, every fart, every episode of vomiting, your mind goes to a different place and it is taxing—it is exhausting. Now every lump and every bump, I almost feel like a hypochondriac because cancer in and of itself is such a scary thing when you don’t—we’ve never been faced with anything like this before—it’s a whole new world.”

Thus, it is within this context that the participants shared their experiences in providing care for their child with a LLI or LTI, and during which they explored the KHP Toolkit. Overall, four themes emerged during data analysis including Fostering Emotional Experiences, Living in the Moment, Remembering Self, and Supporting Social Preferences.

#### 3.2.1. Fostering Emotional Experiences 

Fostering Emotional Experiences encompasses the positive and negative thoughts, feelings, and emotions that the participants described. As one explained, “there are so many emotions on this journey that you can’t even anticipate, and sometimes you feel victorious over really trivial things but then you feel really desperate.” While challenging, participants appreciated the chance to recognize these emotions in a private way through the Toolkit. One participant highlighted the need to manage her emotions, saying “its about recognizing what you’re feeling but you don’t have to fix them in that moment. If you’re feeling helpless, overwhelmed, or stressed or whatever, just feel that way for a minute, or ten, without immediately trying to fix it.” 

Feelings of anxiety were common amongst the participants, and difficult to manage, with one parent saying, “I have found more anxiety and worry than I did in the beginning. Almost like a post-traumatic stress type of thing. So it is good to check in with that with the booklet.” Another mum discussed the ways in which she engaged with the KHP Toolkit in order to help calm her anxiety, saying “this is all pretty scary right. I am so anxious but the colouring activity especially, well, I mean, you know, when you are colouring you are not thinking about that right, you are thinking about what colour to choose in there. You know 10–15 min you are just not in it.” Finally, another participant found that she was able to connect with her kids through the use of the KHP Toolkit by engaging in some of the games that were included. The participant stated “I like all those activities of five or 10 min bursts of time with the kids. Hangman or tic-tac-toe that are fun and get your mind off things, ease that anxiety. Kind of quiet the mind and yeah … simple pleasures.”

Stress was another common experience for the participants in this study. One participant stated “this has been five months of being here and being—our family needs to take some time for ourselves to make, like, kind of normal as it can be and handle some of the stress—take care of each other.” Most of the participants used the KHP Toolkit to manage stress by engaging with the journaling pages where there were prompts to use the free space to write down and reflect on their thoughts. As one participant stated “the journaling, the guided questions in the Toolkit, really prompted me to write things down and it helped me to get stuff off my chest that I didn’t even realize I’d been stressed about.” Participants discussed the flexibility of the KHP Toolkit and the ease of taking it to appointments, stays in the hospital, and at home which allowed for use wherever and whenever stress occurred. As one participant said “I would pull it out on days I was feeling stressed and just grab a page and either start doodling or journaling or reading through some of the information and that was good.” Some of the parents took advantage of other activities such as colouring which was found to be a relaxing stress reliever. The mindfulness activities such as the finger labyrinths were also deemed useful, as one participant explained “all of those activities, especially the mindfulness exercises really helped calm yourself in certain moments –even just normal parenting stuff right—being frustrated with my kids—here’s some tips for calming down a bit so I’m not spazzing.”

Hope was a positive experience for participants and one that was connected to their faith. Hope appeared to keep the participants moving forward. As one parent said “I have a faith in God and that really keeps me grounded. But the Toolkit showed me how that faith connects to my hope. It is my main source of hope. This is a good, if you wanna’ call it, touchpoint.” The KHP Toolkit helped parents to remain hopeful through a section about restructuring their hope that included quotes and ideas for activities to keep their hope possible. As one parent said of the KHP Toolkit,

“It helped me to keep my faith up just because anything can happen and miracles that can happen. So, it helped me to have hope for him that he’ll pull through and that things will start working out how they are supposed to.”

Similarly, another participant found that “it has actually helped me when I was on the verge of, like, despair hanging out on the couch all day. I am worried, then the positive reinforcements coming at you, it is like hope.” Ultimately, participants seemed to agree that “there is room for hope, always room for hope and I’m very hopeful but there is also a lot of worry right now.”

Generally, most participants had found ways to cope with the variety of emotions and feelings that they experienced but the KHP Toolkit provided additional strategies to manage when they most needed it. The activities threaded throughout the KHP Toolkit and at the end, such as colouring, space for journaling, and games, provided participants with options that helped to take their minds off of their busy day to day lives. Other participants felt journaling was most helpful in supporting their coping, as exemplified in the following statement,

“With my daughter’s diagnosis I had to decide quickly how I was going to cope with everything. I like thinking of things, like positive things that happened during the day. I tried to do that in my head before bed but it was really nice to have space in the toolkit to put it down on paper and see that—yes there are lots of positive things that are happening.”

Similarly, another participant said “it was pretty good because the toolkit got me thinking about questions that I don’t ask myself. Like just dealing with a sick child you are so distracted half the time you aren’t taking a good look at how you are feeling and you’re coping with everything. So, I liked that.” However, another participant found the journaling to be difficult as evidenced in the following quote “in the beginning it was cathartic but in time, for me, it’s more painful to journal, and almost relive it—I don’t ever want to go back to those days. The writings I did in the beginning, I’ll burn them. I don’t ever—I don’t want to read that.”

Overall, parents found the KHP Toolkit to be a positive resource that supported them in managing the “dark days” and their busy lives by permitting them to reflect on their difficult or negative emotions and feelings, and by supporting their hope and coping. As one participant said “it helps, share it (emotions) with yourself, share it in your journal, with your partner.” While a few participants did not like to journal and one found journaling to be difficult, the majority used the journaling spaces throughout the toolkit and requested that more be included. As one parent said “the toolkit was very helpful. You can take the time, you can write things, you can vent through the book—I just wrote tons of stuff—it is very helpful that way.” The colouring and mindfulness activities, along with the games (hangman, tic-tac-toe) were effective in reflection and finding some peace in the chaos.” 

#### 3.2.2. Living in the Moment 

Participants clearly articulated the loss of control and feelings of powerlessness they experienced at the time of diagnosis and throughout their experiences in caring for their child with a LLI or LTI. As one parent stated, “I felt really powerless because you’re just told ‘be at this appointment’. ‘Do this’. ‘That is what’s happening’.” Additionally, participants often felt overwhelmed with their circumstances and found it difficult to think too far ahead or to set future goals: “If I think too far ahead then I psyche myself out and then I think of all the bad stuff that could happen. Or what not, so I just try to live in the day, in the moment.” Another participant stated “I just live in the moment and you know, enjoy the days and the times that you have. I find that hard sometimes, but just enjoy the small things.”

As one participant stated “it takes time to kinda’ come to terms with everything and get your head wrapped around it. I feel like it’s a journey for our kiddos and a journey for us, too. Different journeys but just to keep in mind that it is the journey and to enjoy all of the little things along the way.” Similarly, participants also discussed the need to live in the moment in order to care for the child as one explained,

“I think you have to guard yourself. There are people who will say ‘you must be so scared, you must be so upset, or you must be doubting your faith.’ Well, I mean there is some of that in the background but I’m trying to stay in the moment, you’re just trying to be strong for your child.”

Another parent suggested,

“I hated saying this, but it is true. But, just breathe and take one day at a time. You can’t plan what you’re going to do next week, or you know. Maybe next week we’ll do this and this and this. We’ll worry about tomorrow and that’s it.”

Thus, using the KHP Toolkit supported parents in living in the moment, an important part of gaining some control, feeling empowered, and keeping hope possible. 

Another way that the KHP Toolkit encouraged participants was through finding a way to create a unique tradition, service, or special event to help their family celebrate successes and acknowledge their unique journey. Participants discussed the ways in which they used the KHP Toolkit in order to live in the moment. One mum said “the toolkit, what I like was the reflective nature that it, it encouraged me to just sit down and think about where I was in the moment. That was painful, and it was helpful, and it was reflective, altogether.” Another participant “liked documenting our milestones, too. We celebrate every little thing she does so I enjoyed documenting that and getting to think about all of those wonderful accomplishments.” Similarly, a mum said “the toolkit helped to remember everyday that there are lots of positives to be seen and not to lose sight of all the little joys in every moment that are in life and that the little kiddos have.” Furthermore, parents enjoyed making sure that the little and not so little things were remembered, cherished, and celebrated. For example, one participant said,

“The page about celebrating every milestone spoke to me the most. Whether it be the end of a treatment phase, or just remembering like a family member’s birthday and stuff still exists everyday outside of treatment. And it is still important to honour those days. That was my favourite page in the book—a good reminder.”

The KHP Toolkit was well-aligned with the participants’ experiences and supported them in a variety of ways and thus, met unique and diverse needs. Simple activities such as focusing on breathing, not planning too far in advance, and finding the positives and joy in every moment helped parents to feel empowered and to keep their hope. As one participant said “the Toolkit showed me how to focus on my breath—just focusing on your breath and stay in the moment. That really then kind of helped bringing you back to—just settling.”

#### 3.2.3. Remembering Self

The third theme, Remembering Self, spoke to the importance of remembering to care for oneself, but also the contrary nature of the complex, busy parental role that prevented them from doing so. Speaking to the difficulty in prioritizing self-care, one participant stated,

“I’m not very good at it—I don’t think any parent is. It was nice to read that and feel like ‘yeah, taking care of you is important.’ Unless you make an effort, you don’t do it, right? It helps you be more mindful—like I haven’t done anything for myself lately.”

However, as one mum pointed out, with a sick child self-care is not always easy or realistic,

“You know, like if you have a sick kid, it’s kind of hard to justify going for an hour massage, you can’t. I like that the toolkit asks you to write down a few ideas, ‘like oh yeah, there’s lots of things that help me take care of me that I like to do. Kind of broadened my horizon a little bit because you kind of get—this makes me happy—I can try this.”

In agreement, one parent stated,

“A huge part of self-care was choosing the areas where I could take control and owning those. I think lots of people go to ‘oh, self-care is going for coffee or a massage’ or whatever. But sometimes there is as much mental self-care, and so those reminders that self-care can be more than just luxuries was helpful. I said, when I leave the house, I’m going to make a point of putting on an outfit and not just putting on yoga pants. Braiding my hair instead of the classic messy bun or whatever.”

The same participant went on to say that taking back some power and control was accomplished through other choices,

“I felt like I couldn’t even choose to use cloth diapers (like before her diagnosis), I couldn’t even choose what diapers to put on my kid. So, like now we’ve just had a chemo hole so ‘we’re using cloth diapers this week’ because we can. It’s just those little things but I get to choose—it’s more laundry but I chose this.”

Importantly, the participants realized the effort placed on oneself as being a critical part of being able to care for their child. For example, one participant stated, “everybody always tells me, ‘you gotta eat. You gotta’ sleep. You gotta’ look after you, because if you get sick, who is going to be there for her?’” Another participant said, “we have to be at our best to keep them at their best but we put on our super capes and they never come off.” 

Parents used the KHP Toolkit in an effort to schedule time to prioritize self. As one mum said, “the toolkit helped me to remember that even taking a few minutes here and there. Doesn’t have to be always big. To sit down for a cup of tea for five minutes, if that is all you can do, then that’s—enjoy the little things.” Similarly, another mum said “it’s the little things that I remembered and tried to make a habit. I found a really good tea and 9 o’clock is tea time. Get the kids to bed and that really helped me.” One mum appreciated having to write down what she did to engage in self-care as a way to hold herself accountable. She said “I like that part, too, where you had to write out what you did for self-care because sometimes you have to kind of remind yourself what makes you feel like a person.” 

Accordingly, the KHP Toolkit supported participants in consciously thinking about some of the things that allow them to take a moment in the day to care for themselves by doing some of the long forgotten things that bring organization to daily life, and to remember to make time for the “simple pleasures” like a peaceful moment and time alone. 

#### 3.2.4. Supporting Social Preferences

Participants discussed their need for support with social preferences, whether that meant disengaging to some extent and in some areas, or engaging with a circle of support. There were a variety of experiences and preferences, from participants finding that friends and family demonstrated a fading presence over time, while other participants wanted privacy, and yet others sought social support from friends and family, counselling and social media. As one mum said,

“I find a lot of my friends have kinda’ stepped back instead of stepping up. A lot of the mums have told me very similar that people that you want and pray and hope to be, have stepped back. It happens a lot—they stop calling. Like you become the plague essentially.”

Another participant said that she and her husband felt that something was always going wrong and that their persons of support were getting tired of their sharing bad news. She said “then you think people don’t want to hear, or they’re like ‘oh gosh (she) is calling again, I don’t want to answer the phone’. And, that’s just the feeling you get”. This mum appreciated the list in the KHP Toolkit in which she developed a list of support people, saying “When you can actually think of this as your second person on the list, then it helps a lot actually.”

One mum appreciated the KHP Toolkit because it helped her to identify support persons she felt she could rely on to call when needed. Others agreed about the importance of connecting socially in that it allows for some sense of normalcy, as one mum stated “you are being normal for an hour. It is that easy, it is just a coffee—I wish they knew what I know myself, I worry about ‘oh, I don’t want to say the wrong thing’.” Another participant stated,

“I have an amazing community so people have reached out to us. It’s amazing. I can see that it is hard to reach out. It really is. So having somewhere to write down who you want to connect with and keep informed or reach out to, is really helpful.”

Some participants talked about having to create some distance, socially. The KHP Toolkit helped to put that difficult realization into perspective. She said,

“There are times that you know, even if it’s someone you know cares about you, you have to just kind of maybe just drop some contact with them. You can’t care too much about hurt feelings because you only have so much emotional energy. If someone that you know cares about you is trying but it’s not helping, you might have to hurt their feelings by drooping out of their life—you can explain you just need some space. Surround yourself with people who will respect that, understand it.”

Overall, the KHP Toolkit helped parents to document people who were supportive and could be called when needed to provide specific supports. A participant summarized the support the KHP Toolkit offered by stating, “I wrote down my sister-in-law and my mum and my sister. Their phone numbers and email and stuff. It was nice to see how much people I have to help me and what they did, right.” It encouraged the participants to reach out to people who could lend a supportive ear, take time for a coffee, or who they could count on when needed. The KHP Toolkit also allowed participants to visualize the support that they had should they need it. 

#### 3.2.5. Suggested Improvements and Benefits of Participation

The administration of the KHP Toolkit involved a short discussion and demonstration about how to use the KHP Toolkit. The Toolkit also contained a very brief guide for potential uses, but parents were instructed to use it when they had time and to select any of the four sections and eight related activities that felt most appropriate at any given time. While some parents appreciated the flexibility and that there was no pressure to “get it done”, other parents felt the lack of structure and no prescriptive timeframe left room for “procrastination” and the opportunity to set the KHP Toolkit on the bookshelf where it was out of sight and mind. As such, some parents did not use the KHP Toolkit as much as they might have. One parent said “I would have probably done a little bit more in-depth on it if it was more, maybe, concentrated if we had a bit of a deadline, I guess.” Other parents were just too busy during a chaotic time to dedicate time for the KHP Toolkit, as one mum explained “I looked through it but I never actually read it all, just some parts. I was too focused on my son in the hospital so I didn’t have time for it.” To support increased use, some of the participants felt that an electronic version of the KHP Toolkit would be more accessible. As one participant stated “I’m on the phone most of the time or you know, with my children. So the booklet gets put aside- you actually have to remind yourself ‘oh, I should look through the Toolkit’. So, I think like being on the phone would be the best way to go.” Other improvements suggested by participants included making the KHP Toolkit smaller in size so that it was more easily transported, adding more space for journaling, and making it accessible to other family members like grandparents and siblings of the child. For example, one participant would have liked to “involve more family members like grandparents or even my son—it might have helped him work through some of his feelings about what’s going on with his sister.”

Overwhelmingly, participants pointed out their satisfaction in participating in the research, knowing that it could be helpful to themselves or their family. More importantly, participation meant that it may support other families in similar circumstances. As one participant said, “It was a warm feeling to know that there’s something going on and that there’s people trying to gain more research on individuals and families that are going through tough times like this. Yeah, that was really helpful to know that there’s something like this.” The importance of participating in research was evident when another mum stated “’cause we felt pretty hopeless during our journey and if we could shed any light on any part of it for somebody else or some research, we are for that, right?” All participants appreciated the flexibility of the research approach wherein they could choose to complete the measures in person during an appointment or electronically at home, with many choosing to complete them at home on their own time. Participants also responded very positively to the measures, feeling as though they were an excellent way to check in with their current emotional status. As one participant said:

“The questionnaires were easy to answer and evoked a thought process of what’s going on because I think a lot of times with parents—and I have met a lot of parents since I first met with you that are going through similar experiences, and I think a lot of times we suppress our own feelings in order to accomplish the task at hand which is our child’s medical care and we don’t focus so much on the self-care required in order to continue in a long term sense with that medical and also emotional support for the sick child, as well.”

Furthermore, the benefits that were experienced through the use of the KHP Toolkit were most evidenced by the request to provide it earlier in the families’ experience, perhaps at the time of diagnosis. The following quote speaks to the need for the KHP Toolkit early on, “I liked how it was set up. I wish they had given it to us earlier when (my child) was diagnosed. Because some of the stuff I had worked through myself and it would have been good to have that support earlier.” Similarly, another participant stated “I really wished I had it sooner. Cause it was definitely rough for me for the first while to get my head wrapped around everything and this would have been a nice way to walk me through it a little better.” 

## 4. Discussion

The complex nature of families’ experiences of having children with LLIs and LTIs meant that each participant was at a different point in their child’s illness trajectory, as well as their own process of navigating difficult and unexpected circumstances. Participants’ responses to the quantitative questionnaires varied depending on how they and their child were doing that day. Various factors influenced participants’ well-being, and it would not be possible to isolate the intervention as the cause of changes in the four measures. Furthermore, the insignificant results do not mean that the intervention was unsuccessful in positively influencing participant outcomes. As such, it is essential to acknowledge the quasi-experimental design of the study in interpreting the results of the quantitative portion of the study. Along with the design, the participant’ busy lives seemed to negate comprehensive and consistent use of the KHP Toolkit during the research process which may have altered the findings. Notably, our quantitative findings show that parents had a decrease in psychological stress after the interventions which is a new and unique finding. Additionally, the qualitative findings permitted an enhanced understanding of the participants’ experiences and overall thinking about the KHP Toolkit, highlighting the ways in which they used it and found the KHP Toolkit to be a positive psychosocial support in their day to day lives. The four main themes represented the ways in which the participants felt supported in their caregiving for a child with a LLI or LTI, providing a unique and novel addition to the existing literature. Together, the quantitative and qualitative findings indicate that use of the KHP Toolkit influenced family caregivers’ wellbeing, particularly in terms of distress, along with development and implementation of practical and emotional coping strategies.

### 4.1. Fostering Emotional Experiences

Our findings are consistent with a number of studies that have explored the diverse, dynamic, and complex emotions that families endure when caring for children with LLIs and LTIs including anxiety, distress, stress, hope, and coping [3,11,13,29]. The participants who engaged in the current research described ongoing feelings of anxiety, stress, and distress which were most intense at the time of diagnosis but persisted throughout treatment. The participants also indicated that coping with these feelings was often challenging, and that hope was important in helping them to engage in daily caregiving activities, but sometimes difficult to maintain. 

A few existing interventions for parental caregivers provide supporting evidence of the importance of hope in parents’ lives when caring for a child with a LLI or LTI. For example, Fung et al. (2011) [14] and Fonesca et al. (2021) [29], revealed that hope plays a significant role in parents’ ability to face challenges and participate more effectively in parenting. In their interventional pilot studies, a strength-focused parent support program and the administration of therapeutic letters (respectively) were shown to effectively enhance hope in parents caring for their children with cerebral palsy and chronic complex conditions. Similarly, while ongoing, Ho et al.’s (2020) [17] study revealed that their NeW-I intervention, a web-based, therapist-facilitated, strength-focused, and meaning-oriented intervention for parents of children with chronic life-threatening illnesses shows promise in supporting and enhancing parents’ sense of hope. 

Thus, similar to existing interventional studies aimed at supporting family care [13,14,15,16,29], our findings demonstrated positive reception for the KHP Toolkit intervention. Following the use of the KHP Toolkit levels of psychological distress were significantly lower at each time point (Time 2, 3 and 4) when compared to pre-intervention levels. Although not statistically significant, data analysis showed that the participants had increased hope and self-efficacy and decreased uncertainty after the intervention at all time points post-intervention. Additionally, participants provided qualitative evidence indicating the positive support the KHP Toolkit provided in their daily lives. The KHP Toolkit fostered management of emotional experiences through coloring activities, games, journaling which helped participants to manage the “dark days”. As well, it was seen as a positive resource to promote hope and coping, and management of anxiety and stress. As such, the KHP Toolkit is novel in that it provides a flexible, easy to use, convenient, adaptable, and comprehensive approach that healthcare providers can use to assist family caregivers in navigating a multitude of emotional experiences as they care for and support their children with diverse LLIs and LTIs. However, additional refinement of the KHP Toolkit will be completed based on participant feedback including more space for journaling, increasing the size of the calendar for organization of daily life activities, translating to commonly used languages such as French, Dene and Cree (two Indigenous languages used in the region). Future research will also focus on enhancing accessibility of the KHP Toolkit through digital applications for use with family caregivers. 

### 4.2. Living in the Moment

Living in the Moment was described by participants as a means to manage feelings of powerlessness, loss of control, and being overwhelmed during their experience of caring for their child and managing daily life as a family. Participants strove to live in the moment by making even the smallest of choices such as which diapers to use for their child when possible, what to wear when going out, and abstaining from planning too far ahead. Other studies offer similar insights into how parental caregivers consciously strategize in order to remain in the present and not think too far into the future to avoid feelings of being overwhelmed [19,30,31]. 

In the current study, the participants found that the KHP Toolkit acted as a reminder and supported critical strategies to Live in the Moment including breathing exercises, journaling, and celebrating all milestones along the way. In support of the idea that celebrating milestones is important to family caregivers, the qualitative study completed by Fonseca et al. (2021) [29] found that parents of children with chronic complex illnesses would have appreciated therapeutic letters at times that would have encouraged them to celebrate successes and victories. These participants stated that this would have sustained their continued caregiving activities. While this theme highlights a strategy that is commonly identified in the literature, to our knowledge the KHP Toolkit is the only psychosocial intervention that specifically supports caregivers of children with LLIs and LTIs to live in the moment in order to keep their hope possible. In doing so, participants, described feeling more empowered, in control, and less overwhelmed, if even for a short time. To address this gap in the literature and to build a more defined base of evidence from which to support parental caregivers, additional research is needed to explore the feasibility of mindfulness activities such as breathing and journaling, and examine the influence of such activities on loss of control, powerlessness, and feelings of being overwhelmed. 

### 4.3. Remembering Self

Remembering self is a critical aspect of caring for children with LLIs or LTIs as the participants in this study made the direct connection between their own health and the health and wellbeing of their child. The importance of self-care was central to this notion, yet was at odds for some participants who acknowledged the importance of self-care but also challenges related to prioritizing such care given the demands of caregiving for children with complex care needs. For the participants in this study, remembering self meant prioritizing oneself in order to participate in self-care activities that were associated with improved health and wellbeing. For some participants, this meant “simple pleasures”, relaxation, and taking time for self in a variety of different ways. 

Traditional ideas about self-care, including those strategies suggested by their social circle like massages and nights out with a partner, were often not possible for participants in this study. Most participants were reluctant to leave their child even with a close family member, which meant that many options for remembering self were not appropriate. However, participants stated that the KHP Toolkit supported them in thinking more broadly about self-care and cued them to think about manageable activities to help with relaxation and calming such as a nightly cup of tea and recalling things that once made them happy. Participants noted that such strategies should be re-established as habits that can easily be maintained from day to day. While none of the existing pediatric support interventions focused on self-care, some of the parents who engaged in Lindenfelser et al.’s (2012) [15] music therapy intervention found it relaxing. Thus, the activities presented in the KHP Toolkit like finger labyrinths, coloring, and reflective strategies are novel, well-used by the participants, and were noted to be those things that could be done anywhere and at any time, an aspect of the KHP Toolkit that is unique and not necessarily found in other pediatric support interventions. 

### 4.4. Supporting Social Preferences

Due to wide variation in the ways in which participants experienced and wished to engage with their social circles, many had differing preferences requiring supportive care. For example, some participants described friends and family members who seemed uncomfortable or awkward with socializing, while others had distanced themselves, leaving participants feeling like “the plague.” Similarly, some participants wished to reach out and create a group of supporters and people they could connect with including other parents in similar circumstances, friends, family, and formal counselling. Other participants engaged with social media to share their stories through Facebook and personal blogs, and to connect with others to learn from their experiences and share their own. However, a number of participants wanted space and solitude to connect and care for their immediate family without the guilt of hurting others’ feelings or spending energy ‘hosting’. Similar to the findings in this study, existing research highlights the variation in social support needs of parental caregivers of children with LLIs and LTIs [3,13,19,32]. Additionally, four of the existing pediatric supportive interventions noted improvements in the social dimension of health following administration. For example, the interventions developed by Noyes et al. (2013) [18] and Fonesca et al. (2021) [29] provided participants with feelings of support from their healthcare team. Using music therapy, Lindenfelser et al. (2012) [15] found that the parents in their study enjoyed and benefitted from participating in the music session together as a family. Similarly, Fung et al. (2011) [14] found that when parents completed the strength-based focus groups, they experienced lower levels of feelings of social isolation. While each of these interventional studies influenced different areas of social engagement, the findings underline the importance of facilitating social engagement for parental and family caregivers. The KHP Toolkit appears to be unique in its individualized, focused, yet broad approach to supporting parents through identification, documentation, and visualization of favored strategies for social interaction. 

While the existing literature and, more specifically, the abovementioned interventions [14,15,16,17,18] show promise in supporting dimensions of psychosocial well-being in parents of children with LLI and LTI, some are focused on a specific parent population (e.g., parents of children with cerebral palsy, cancer, or terminal illness), leaving few instances of interventions that are broadly applicable to parents and other family caregivers of children with diverse LLI and LTI. Additionally, participation in certain existing interventions required parents to attend in person and/or have children who are currently receiving in-patient care, which can minimize accessibility. Accordingly, some family caregivers will appreciate supportive tools that are flexible and convenient to access, such as the KHP toolkit which is self-administered and flexible. Family caregivers can use the KHP Toolkit when their child is an in- or out-patient, including while at home, and in conjunction with more formal support programs if desired. The KHP Toolkit is comprehensive in that it is focused on many aspects of caregiver health and wellbeing including diverse feelings, emotions, and experiences including hope, living in the moment, self-care, and social preferences. As such, it can be easily administered with wide application to diverse parental needs and preferences.

## 5. Strengths and Limitations

There are strengths and limitations related to the design of this study. For example, the respondents remaining at Time 4 may been those with higher or lower levels of distress, self-efficacy, uncertainty, and stress. Therefore, there was potential bias given their responses. Confidentiality was maintained at all times; however, there was the possibility that participants discussed the study with one another and, therefore, there was a risk of cross contamination between the two groups, although this was unlikely given the lengthy recruitment and data collection period, and recruitment of parents of children who were in- and out-patients. Additionally, the sample was made up of mainly Caucasian mothers who were married which may have limited other perspectives; however, some diversity existed in terms of gender, age, location of residence, and ethnicity. These factors along with the attrition at Times 3 and 4 may preclude generalizability of the study findings. While, statistically significant findings were reported, a larger sample size may have supported the determination of significant relationships amongst other variables. However, a major strength of the study was the crossover design, randomization of participants, the use of both qualitative and quantitative data, and the longitudinal nature of data collection. The findings contribute to the development of enhanced supportive care for parents of children with LLIs and LTIs and inform direction for future research.

## 6. Conclusions

All participants recognized the critical nature of their own health and wellbeing in relation to that of their child’s and, therefore, appreciated the opportunity to use the flexible, easy to understand, self-administered KHP Toolkit. Unfortunately, the complexity of their busy lives prevented in-depth and consistent use of the KHP Toolkit for some participants during participation in this research. The fragility and precarious nature of their child’s health added to the participants’ ever-changing experiences, meaning that the KHP Toolkit had varying importance depending on circumstances. Thus, the quantitative findings showed that the KHP Toolkit significantly influenced distress, while changes in hope, self-efficacy, and uncertainty were not significant due to the difficulty of quantitatively capturing the impact of the KHP Toolkit in the diverse sample. However, together, the quantitative and qualitative findings of the study reveal the potential of the KHP Toolkit in supporting parents’ psychosocial wellbeing as they navigate the complexities of the care and support of children with LLIs and LTIs. Furthermore, the unique dimensions of this intervention and the findings add to existing literature on psychosocial support interventions for this population. As well, the findings provide a distinct foundation for future research with a refined KHP Toolkit aimed at: engaging other family members; examining the potential for translation to other languages and into a digital format; and, evaluating a refined KHP in a large national sample of family caregivers. 

## Figures and Tables

**Table 1:** Description of the Variables.

Variable	Number of Items	Cronbach’s Alpha Pre and Post Intervention	
Herth Hope Index (HHI)	12	0.86	0.87
Parental Perception of Uncertainty Scale (PPUS)	31	0.91	0.93
General Self Efficacy Scale (GSES)	10	0.82	0.81
Kessler 6 Psychological Scale (K6 Distress)	6 *	0.83	0.80

* This measure asks about 6 different feelings of distress as experienced over the past 30 days, followed by 5 questions about frequency of feelings or other causes that offer context to the level of distress reported and can be analyzed separately.

**Table 2:** Participant Demographic Characteristics.

	Group 1 *n*	Group 2 *n*	Total *n*
**Gender**			
Male	6	6	12
Female	23	23	46
**Age of Parent/Guardian**			
20–29 years	6	11	17
30–39 years	14	11	25
40+ years	9	7	16
**Parent/Guardian Education**			
Less than High School/High School	10	11	21
Vocational College	11	7	18
Bachelors Degree/Post Graduate	8	11	19
**Marital Status**			
Married/Common Law	24	24	48
Divorced/Separated/Widowed/Single	5	5	10
**Ethnicity**			
Caucasian	18	21	39
Visible Minority	4	2	6
Indigenous	7	6	13

**Table 3:** Comparison of the Means.

	Herth Hope Index (HHI)	Parental Perception of Uncertainty (PPUS)	Self-Efficacy (GSES)	* Psychological Distress (K6)
Means: T1 (*n* = 58)	37.71	89.67	32.29	8.54
Means: T2 (*n* = 50)	37.04	90.58	31.83	6.25
Means: T3 (*n* = 45)	36.67	88.25	31.54	6.38
Means: T4 (*n* = 26)	37.96	87.21	32.58	5.79

* K6 F = 6.407 (*p* = 0.001).

## Data Availability

Study data were collected and managed using REDCap electronic data capture tools hosted at University of Saskatchewan [33]. REDCap (Research Electronic Data Capture) is a secure, web-based application designed to support data capture for research studies, providing (1) an intuitive interface for validated data entry; (2) audit trails for tracking data manipulation and export procedures; (3) automated export procedures for seamless data downloads to common statistical packages; and (4) procedures for importing data from external sources. The data are not available to the public.
